# Local Effects of Short‐Term Ovum Pick‐Up Exposure on Ovarian Follicular Dynamics and Extracellular Matrix‐Related Gene Expression in Pre‐Pubertal and Pubertal Heifers

**DOI:** 10.1111/rda.70332

**Published:** 2026-11-13

**Authors:** Sandra Gąsiorowska, Izabela Wocławek‐Potocka, Dorota Boruszewska, Ilona Kowalczyk‐Zieba

**Affiliations:** ^1^ Embryo Biology Team InLife Institute of Animal Reproduction and Food Research, Polish Academy of Sciences Olsztyn Poland

**Keywords:** extracellular matrix, follicular regulation, ovarian follicles, ovum pick‐up (OPU)

## Abstract

Follicle activation is a key step in folliculogenesis, regulating the recruitment of follicles into the growing pool. Local ovarian factors, including the extracellular matrix (ECM) and the ovarian microenvironment, may contribute to the regulation of follicular dynamics. This study investigated the effects of short‐term ovum pick‐up (OPU) on follicular dynamics and the expression of genes related to ECM remodelling and follicular regulation in pre‐pubertal and pubertal Holstein–Friesian heifers. Fourteen heifers (seven pre‐pubertal and seven pubertal) underwent five OPU sessions. The right ovary was subjected to repeated puncture, whereas the left ovary served as an internal control. Follicle number and ovarian area were assessed by ultrasonography, while circulating concentrations of oestradiol (E2) and anti‐Müllerian hormone (AMH) were measured. The expression of genes related to ECM remodelling (*COL3A1, HAS3, MMP2, TIMP2*) and follicular regulation (*PTEN, KITLG, HSD17β11*) was analysed using quantitative real‐time PCR. Follicle number and ovarian area were analysed using three‐way repeated‐measures ANOVA, hormone concentrations using repeated‐measures ANOVA, and gene expression using two‐way ANOVA, with Tukey's post hoc test applied where appropriate. A transient increase in follicle number was observed in the punctured ovary during the fourth OPU session in both pre‐pubertal and pubertal heifers. No significant changes were detected in ovarian area or systemic E2 and AMH concentrations. Short‐term OPU exposure was associated with changes in the expression of genes related to ECM remodelling and follicular regulation in the punctured ovary. These responses differed according to pubertal status, suggesting developmental differences in the local ovarian response to repeated puncture. Overall, under the experimental conditions of the present study, five OPU sessions were associated with local changes in follicular dynamics and ovarian gene expression, with responses differing according to pubertal status.

## 1 Introduction

The activation of primordial ovarian follicles is a crucial step in folliculogenesis, determining the potential for further oocyte development (Shah et al. 2018). While molecular and hormonal signals are well‐established regulators of follicle activation, increasing evidence indicates that the physical properties of the ovarian cortex and extracellular matrix (ECM) also contribute to the regulation of follicle activation and growth (Fiorentino et al. 2023; Shah et al. 2018; Vasse et al. 2025). The structural organization of the ovary and local differences in tissue stiffness may influence follicle activation and growth by shaping the follicular microenvironment (Fiorentino et al. 2023; Ouni et al. 2020; Shah et al. 2018; Vasse et al. 2025). ECM composition also varies with developmental stage, suggesting that sexual maturity may influence the structural and mechanical properties of the ovarian matrix and consequently follicle activation and growth (Ouni et al. 2020). Pre‐pubertal heifers have a less active reproductive endocrine environment than pubertal animals (Atkins et al. 2013), providing a useful model for investigating local ovarian responses to repeated follicular puncture under reduced gonadotropic stimulation.

The ovarian ECM is a dynamic network of proteins, proteoglycans, and glycosaminoglycans that provides structural support and contributes to the regulation of follicle development and tissue homeostasis (Parkes et al. 2021). In bovine ovaries, collagen type III (COL3A1) is the predominant collagen, contributing to follicular wall integrity and tissue stiffness, which helps maintain primordial follicle dormancy (Parkes et al. 2021). Hyaluronan, synthesized by hyaluronan synthases including HAS3, contributes to tissue hydration and ECM organization (Parkes et al. 2021). ECM remodelling is regulated by matrix metalloproteinases and their tissue inhibitors, including MMP2 and TIMP2, which coordinate ECM turnover and maintain tissue homeostasis during follicular development (Lussier et al. 2017; Peralta et al. 2017). In addition to ECM‐related genes, follicle activation and function are regulated by intracellular signalling and follicle‐associated factors, including PTEN, a negative regulator of the PI3K/AKT pathway (Maidarti et al. 2019), and KITLG, which promotes follicle survival and activation (Lussier et al. 2017). HSD17β11 has also been implicated in steroid metabolism during follicular development (Yang and Fortune 2015).

We hypothesized that five OPU sessions would be associated with local alterations in ovarian follicular dynamics and gene expression, and that the magnitude and pattern of these responses would differ between pre‐pubertal and pubertal heifers. Accordingly, the study aimed to characterize the local ovarian response to five OPU sessions by monitoring follicle numbers and assessing the expression of selected genes associated with ECM remodelling, follicular regulation, and ovarian function in repeatedly punctured and contralateral control ovaries. Five OPU sessions were performed on the right ovary at 7‐day intervals, while the left ovary remained unpunctured and served as a within‐animal control. This paired‐ovary design enabled the assessment of local ovarian responses while reducing inter‐animal variability. By comparing pre‐pubertal and pubertal heifers under the same experimental conditions, the present study provides insight into the local follicular and transcriptional responses associated with a short‐term, five‐session OPU protocol at different stages of sexual maturation.

## 2 Materials and Methods

### 2.1 Animals

The in vivo study was conducted in accordance with relevant regulations and guidelines, including Directive 2010/63/EU of the European Parliament and of the Council on the protection of animals used for scientific purposes (22 September 2010), the Polish Parliament Act on Animal Protection (21 August 1997, Dz.U. 1997 No. 111, item 724, as amended), and the Polish Parliament Act on the Protection of Animals Used for Scientific or Educational Purposes (15 January 2015, Dz.U. 2015, item 266). All procedures were approved by the Local Animal Care and Use Committee of Olsztyn (Agreement No. 55/2023). The 14 Polish Holstein–Friesian heifers included in the study originated from a single commercial dairy farm located in Orneta, Poland. Animals were divided into two groups according to age and reproductive status: pre‐pubertal heifers (*n* = 7; 8.4 ± 0.5 months of age; 304 ± 43 kg body weight; BCS 3.0 ± 0.25) and pubertal heifers (*n* = 7; 16.5 ± 0.5 months of age; 460 ± 75 kg body weight; BCS 3.0 ± 0.25). No hormonal stimulation or synchronization of ovarian activity was applied before or during the experimental period. Thus, follicular development and ovarian responses were assessed under physiological, unstimulated conditions.

Animals were classified as pre‐pubertal or pubertal based on age and ovarian reproductive status. Pre‐pubertal heifers were characterized by the absence of ovarian cyclicity, including the absence of a corpus luteum and a dominant follicle. Pubertal heifers exhibited ovarian cyclicity, confirmed by the presence of a corpus luteum and/or a dominant follicle during transrectal ultrasonographic examination. Ovarian status was evaluated immediately before each OPU session.

### 2.2 Ovum Pick‐Up Procedure

Ovaries in pre‐pubertal and pubertal heifers were visualized using a Dramiński 4Vet Slim ultrasound scanner (Dramiński S.A., Olsztyn, Poland) equipped with an 8‐MHz microconvex probe fitted into a custom‐made intravaginal OPU probe holder. Before each OPU session, both ovaries were examined by transrectal ultrasonography. The maximum longitudinal length and transverse width of each ovary were measured directly on the ultrasonographic image in the largest visualized ovarian section. Ovarian area (cm^2^) was subsequently calculated using the ellipse formula: area = π × (length/2) × (width/2). The same measurement procedure was applied to both the right experimental ovary and the left control ovary during each experimental session.

The right ovary was designated as the experimental ovary and subjected to follicular puncture, whereas the contralateral left ovary remained unpunctured and served as the within‐animal control. All animals underwent five OPU sessions at 7‐day intervals using the same procedure. During each session, all follicles ≥ 2 mm in diameter identified by ultrasonography were punctured and aspirated. Follicular aspiration was performed using an 18‐gauge disposable hypodermic needle connected to a 50‐mL conical tube (BD Falcon, BD Biosciences) via Teflon tubing (Cook Veterinary Products). Negative pressure was applied using a vacuum aspiration pump (GAST), with the aspiration vacuum adjusted to obtain a flow rate of 15 mL PBS/min.

### 2.3 Ovarian Biopsy Procedure

For ovarian biopsy collection, the ovaries were visualized using the same 8‐MHz microconvex ultrasound transducer fitted adjacent to a needle‐guidance system in a commercially available OPU handle. To avoid interference with the biopsy procedure, follicles with a diameter of > 8 mm were aspirated using the conventional OPU system before biopsy collection.

The conventional OPU needle carrier was subsequently replaced with a modified carrier equipped with a 14‐gauge disposable needle (38 mm length; 2.1 mm outer diameter), which served as a trocar. A 60‐cm disposable true‐cut biopsy needle (Quick‐Core Biopsy Needle), equipped with an inner stylet, a 20‐mm‐long and 2‐mm‐wide specimen notch, an outer cutting cannula, and an automated spring‐loaded handle with a trigger, was used to collect ovarian tissue.

The 14‐gauge trocar needle was advanced through the vaginal wall, after which the biopsy needle was introduced through the trocar into the ovarian stroma. Before insertion, the biopsy needle was prepared by retracting the plunger and locking the spring mechanism in the ready position. Once the needle was positioned within the ovarian tissue, the inner stylet was advanced to expose the specimen notch, allowing tissue to enter the notch. The cutting cannula was then released to excise the tissue specimen.

A single biopsy insertion was performed in each sampled ovary, yielding one biopsy specimen of approximately 20–25 mg. Each specimen was processed individually and treated as a separate sample originating from a specific ovary of a given animal. Tissue from different animals or from the right and left ovaries was never pooled.

### 2.4 Hormone Concentrations

During each of the five OPU sessions, blood samples were collected from the coccygeal vein to monitor the endocrine status of the animals. Blood was collected using a BD Vacutainer Precision Glide 21G × 38 mm needle (BD, Franklin Lakes, NJ, USA; Ref. 360213) connected to a tube holder, into 9‐mL Vacuette serum tubes with a clot activator (Greiner Bio‐One GmbH, Kremsmünster, Austria; Ref. 455092). Following blood collection, the samples were allowed to clot and were subsequently centrifuged at 1500 × *g* for 10 min at room temperature. The obtained serum was separated and stored at −21°C until hormone analysis.

Serum AMH concentrations were determined by LABOKLIN Polska Sp. z o.o. (Warsaw, Poland) using the Elecsys AMH Plus assay (Roche Diagnostics, Switzerland), an electrochemiluminescence immunoassay (ECLIA), performed on a Roche cobas 8000 analyser. The assay was applied to bovine serum samples. According to the manufacturer, the analytical measuring range of the assay was 0.01–23 ng/mL, with a minimum detection limit of 0.01 ng/mL. The manufacturer‐reported intra‐assay coefficient of variation (CV) was 2.7%–4.4%, and the inter‐assay CV was 1.0%–1.8%.

Serum E2 concentrations were determined at the Municipal Hospital Complex in Olsztyn, Poland, using a Cobas e 411 automated immunoassay analyser (Roche Diagnostics, Mannheim, Germany). E2 concentrations were measured using the Elecsys Estradiol III electrochemiluminescence immunoassay (ECLIA; Cat. No. 06656021190). According to the manufacturer, the analytical measuring range of the assay was 5–3000 pg/mL, with a limit of detection of 5 pg/mL and a limit of quantitation of 25 pg/mL. The intra‐assay coefficient of variation (CV) was ≤ 3.3%, and the inter‐assay CV was ≤ 4.7%, as reported by the laboratory.

### 2.5 RNA Isolation

Total RNA was extracted from ovarian tissue samples collected from pre‐pubertal and pubertal heifers. For each age group, tissue samples were collected from the right ovary during the first and fifth OPU sessions, and from the contralateral left ovary during the fifth OPU session. Immediately after biopsy collection, tissue samples were placed in a PBS‐based suspension medium containing EDTA, prepared according to the manufacturer's recommendations. Approximately 20–25 mg of ovarian tissue was obtained from a single biopsy and processed individually using the PicoPure RNA Isolation Kit (Thermo Fisher Scientific, USA) according to the manufacturer's instructions. Samples were not pooled between animals or ovaries. RNA was purified using spin columns, followed by washing steps and elution in 20 μL of elution buffer. RNA quantity and purity were assessed using a DeNovix spectrophotometer.

### 2.6 Complementary DNA (cDNA) Synthesis

Reverse transcription of RNA to cDNA was performed using the SuperScript III First‐Strand Synthesis SuperMix for qRT‐PCR (Thermo Fisher Scientific, USA) according to the manufacturer's instructions. Each reaction was carried out in a total volume of 21 μL, consisting of 2× RT Reaction Mix, RT Enzyme Mix, RNase H, and RNA (adjusted to 20 ng/μL), with DEPC‐treated water used to complete the volume. Reactions were incubated in a BIO‐RAD T100 thermal cycler at 25°C for 10 min, followed by 50°C for 30 min and 85°C for 5 min. Samples were then chilled on ice. Negative controls without reverse transcriptase were included to assess genomic DNA contamination.

### 2.7 Quantitative Real‐Time PCR

Real‐time PCR was performed using a QuantStudio 6 Flex Real‐Time PCR System and Maxima SYBR Green/ROX qPCR Master Mix (#K0222; Thermo Scientific, USA). Primers were designed using NCBI Primer‐BLAST (https://www.ncbi.nlm.nih.gov/tools/primer‐blast/), and their sequences are listed in Table 1. Thermal cycling conditions included initial denaturation at 95°C for 10 min, followed by 40 cycles of 95°C for 15 s and 60°C for 60 s. A melting curve analysis was performed to confirm amplification specificity. All reactions were conducted in duplicate. Gene expression levels were normalized to *GAPDH*, selected based on its stable expression in ovarian tissue samples. Relative expression was calculated using the ΔΔC_t_ method, and fold changes were determined by comparison with control samples. Relative quantification was also supported by the real‐time PCR Miner algorithm (Zhao and Fernald 2005).

**TABLE 1:** Characteristics of the primers designed for target genes in PCR.

Gene symbol	Gene name	Primer sequence 5′–3′	GenBank no.
*COL3A1*	Collagen Type III Alpha 1 Chain	Forward 5′‐CCCAACCCTGAAATCCCGTT‐3′ Reverse 5′‐GACCATTAGGAGGGCGAGTG‐3′	NM_001076831.1
*GAPDH*	Glyceraldehyde‐3‐phosphate dehydrogenase	Forward 5′‐CACCCTCAAGATTGTCAGCA‐3′ Reverse 5′‐GGTCATAAGTCCCTCCACGA‐3′	NM_001034034.2
*HAS3*	Hyaluronan Synthase 3	Forward 5′‐CTACAAGCAGGTCCATTTGCC‐3′ Reverse 5′‐TTCACTGCTCACCACAAAGGT‐3′	XM_015475704.3
*HSD17β11*	Hydroxysteroid (17‐beta) dehydrogenase 11	Forward 5′‐AAGAGAAAATCAGTCACCGGAG‐3′ Reverse 5′‐TGGCTGTTTCCTCAAGTCC‐3′	NM_001046286.2
*KITLG*	KIT Ligand	Forward 5′‐CGGGATGGACGTTTTGCCTA‐3′ Reverse 5′‐GTCCAGAAGATCAGTCAAGCTG‐3′	NM_174375.2
*MMP2*	Matrix Metalloproteinase 2	Forward 5′‐TCTTCGCCGGAGACAAATTCTGGA‐3′ Reverse 5′‐ATCCAGGTTATCAGGGATGGCGTT‐3′	NM_174745.2
*TIMP2*	Tissue Inhibitor of Metalloproteinases 2	Forward 5′‐CCCATCAAGCGGATTCAGTAT‐3′ Reverse 5′‐ACTCCTTCTTTCCTCCAATGTC‐3′	NM_174472.3

**FIGURE 1:**
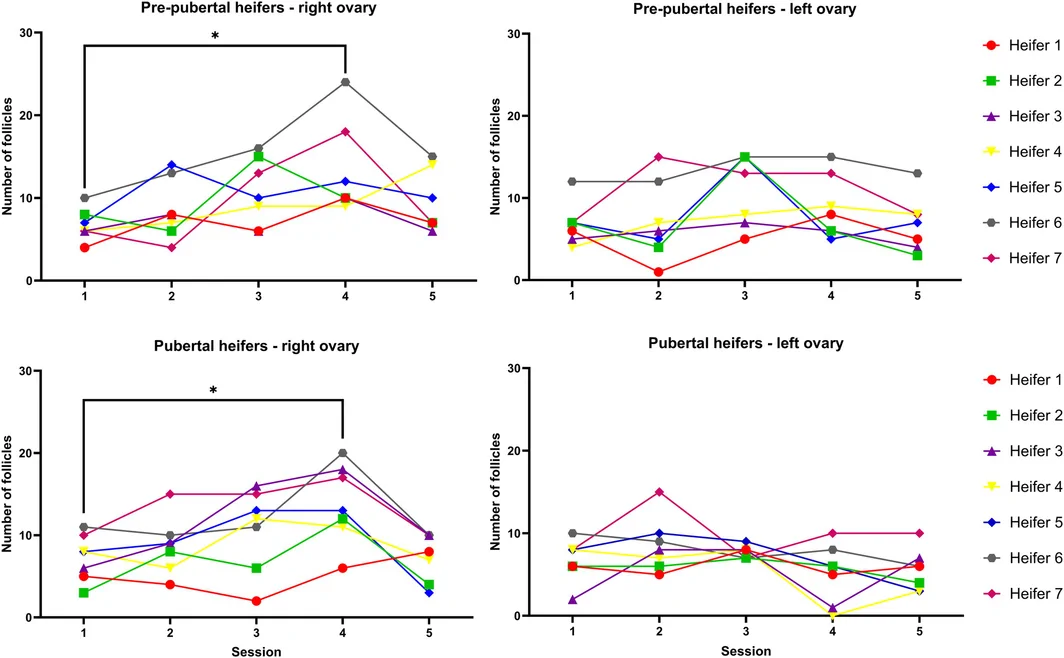
Individual changes in follicle number across five experimental sessions (I–V) in pubertal and pre‐pubertal heifers. Each line represents an individual heifer (*n* = 7 per group). Asterisks indicate statistically significant differences (*p* ≤ 0.05).

### 2.8 Statistical Analysis

All statistical analyses were performed using GraphPad Prism 10 (GraphPad Software, San Diego, CA, USA). All individual measurements obtained from each heifer were included in the statistical analysis without prior averaging, ensuring that the analyses fully reflected experimental variability. Normality of distribution was assessed using the Shapiro–Wilk test. Ovarian follicular characteristics (follicle number and ovarian area) were analysed using three‐way repeated‐measures ANOVA, with experimental session (I–V) and ovary (R vs. L) as within‐subject factors and pubertal status (pre‐pubertal vs. pubertal) as a between‐subject factor. When significant main effects or interactions were detected, Tukey's post hoc test was applied for pairwise comparisons. Hormone concentrations were analysed using repeated‐measures ANOVA. Gene expression was analysed using the ΔΔC_t_ method and two‐way ANOVA with Tukey's post hoc test. A *p*‐value < 0.05 was considered statistically significant.

## 3 Results

### 3.1 Follicle Counts

Differences in the number of ovarian follicles were observed between sessions and between ovaries (*p* < 0.05) (Table 2). Post hoc Tukey's multiple comparisons test showed an increase in follicle number in the punctured ovary during Session IV compared with Session I in both pre‐pubertal (*p* = 0.0331) and pubertal (*p* = 0.0331) heifers. In pubertal heifers, the punctured ovary in Session IV contained more follicles than the contralateral ovary within the same session (*p* = 0.0007), as well as compared with the same ovary in Session V (*p* = 0.0409). No further differences were found between sessions. Individual data points and mean values are presented in Table 2.

**TABLE 2:** Ovarian area and follicle number in right and left ovaries during repeated OPU sessions.

Session	Pre‐pubertal			Pubertal		
	Ovary	Area (cm^2^), mean ± SD	Follicles, mean ± SD	Ovary	Area (cm^2^), mean ± SD	Follicles, mean ± SD
1	*R*	2,08 ± 1,19	6,71 ± 1,89^aα^	*R*	2,3 ± 0,67	7,29 ± 2,81^aα^
	*L*	1,63 ± 0,42	6,86 ± 2,54^aα^	*L*	1,94 ± 1,17	6,86 ± 2,54^aα^
2	*R*	2,05 ± 1,28	8,57 ± 3,64^aα^	*R*	2,13 ± 1,43	8,71 ± 3,45^aα^
	*L*	1,68 ± 0,98	7,14 ± 4,81^aα^	*L*	2,83 ± 1,16	8,57 ± 3,31^aα^
3	*R*	2,05 ± 1,07	10,71 ± 4,07^aα^	*R*	3,25 ± 1,6	10,71 ± 5,02^aα^
	*L*	2,72 ± 1,1	11,14 ± 4,34^aα^	*L*	3,79 ± 0,87	7,71 ± 0,76^aα^
4	*R*	1,8 ± 1,08	13,29 ± 5,62^bα^	*R*	2,78 ± 1,47	13,86 ± 4,81^bβ^
	*L*	1,82 ± 0,64	8,86 ± 3,8^aα^	*L*	2,3 ± 1,07	5,14 ± 3,58^aα^
5	*R*	2,11 ± 1,41	9,42 ± 3,69^aα^	*R*	3,7 ± 1,75	7,43 ± 1,72^aα^
	*L*	1,62 ± 0,57	6,86 ± 3,34^aα^	*L*	3,53 ± 1,68	5,57 ± 2,51^aα^

*Note:* Data are presented as mean ± standard deviation (SD) (*n* = 7 pubertal and *n* = 7 pre‐pubertal heifers). Ovarian area (cm^2^) and follicle number were assessed across five OPU sessions (I–V). Different lowercase letters (a, b) indicate differences in follicle number between sessions within the same ovary, while different Greek letters (α, β) indicate differences between ovaries within the same session (*p* ≤ 0.05). No significant differences were detected in ovarian area. Across five sessions, follicle numbers varied in both groups, showing high individual variability. A transient increase occurred only in the punctured ovary during Session IV, with no change in the control ovary (Figure 1).

### 3.2 Hormone Concentrations

No differences were observed in serum AMH and E2 concentrations between pre‐pubertal and pubertal heifers at any of the analysed time points (*p* > 0.05). No effects of pubertal status or OPU session, or interactions between these factors, were observed for serum AMH and E2 concentrations (*p* > 0.05). Serum AMH and E2 concentrations during the five OPU sessions in pre‐pubertal and pubertal heifers are presented in Figure 2.

**FIGURE 2:**
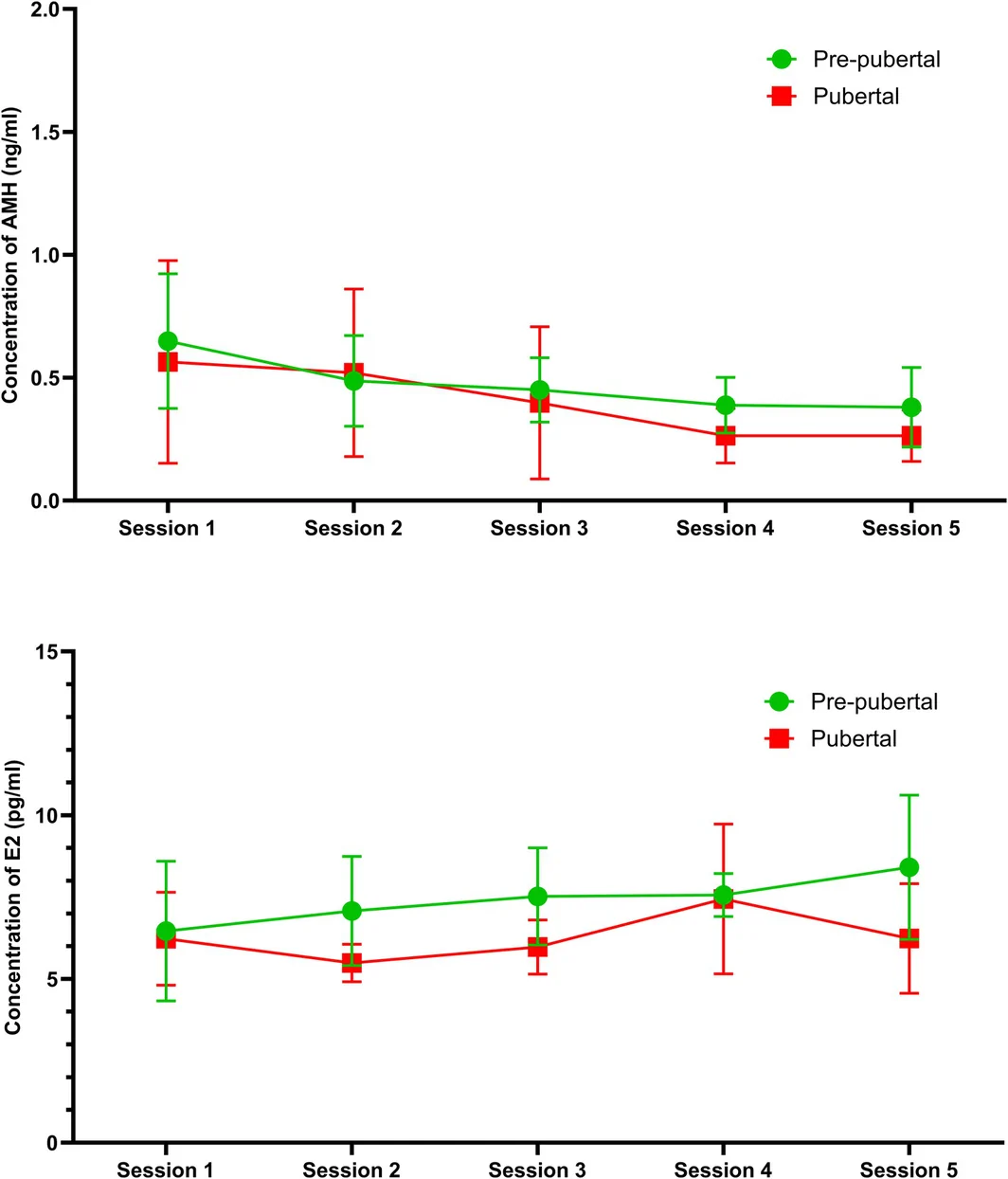
Serum concentrations of AMH and E2 during five OPU sessions in pre‐pubertal and pubertal heifers. Serum AMH and E2 concentrations remained relatively stable throughout the experimental period in both groups. No differences were detected across sessions or between groups (*p* > 0.05). Data are presented as mean ± SEM.

### 3.3 Expression of Genes

#### 3.3.1 Genes Involved in Extracellular Matrix Remodelling

The expression of *COL3A1* differed among the experimental conditions. In pre‐pubertal heifers, *COL3A1* expression in the right (punctured) ovary at the fifth OPU session was higher than at the first session and than in the left (control) ovary at the fifth session (both *p* < 0.0001), whereas no difference was observed between the right ovary at the first session and the left ovary at the fifth session (*p* = 0.6828). In pubertal heifers, *COL3A1* expression in the right ovary at the fifth session was higher than at the first session and than in the left ovary at the fifth session (both *p* < 0.0001), whereas no difference was observed between the right ovary at the first session and the left ovary at the fifth session (*p* = 0.3692). Expression at the first OPU session was lower in pre‐pubertal than in pubertal heifers (*p* < 0.0001), whereas no difference was observed between the right ovaries at the fifth OPU session in the two age groups (Figure 3A).

**FIGURE 3:**
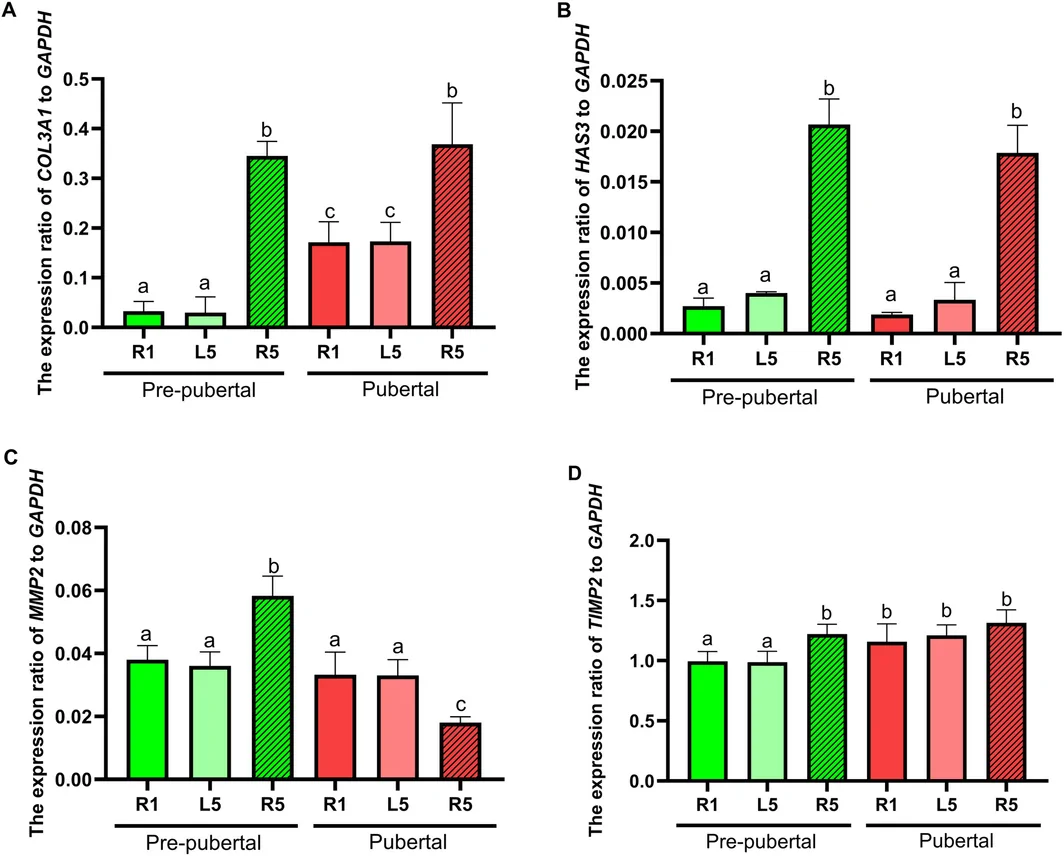
Expression of (A) *COL3A1*, (B) *HAS3*, (C) *MMP2*, and (D) *TIMP2* mRNA in ovarian tissue of pre‐pubertal and pubertal heifers subjected to five OPU sessions. Data are presented as mean ± SEM. Differences between compared conditions are indicated by different lowercase letters (a, b, c; *p* < 0.05). L5, left (control, non‐punctured) ovary at the fifth OPU session; R1, right (punctured) ovary at the first OPU session; R5, right (punctured) ovary at the fifth OPU session.

A similar pattern was observed for *HAS3*. In pre‐pubertal heifers, expression in the right (punctured) ovary at the fifth OPU session was higher than at the first session and than in the left (control) ovary at the fifth session (both *p* < 0.0001), whereas no difference was observed between the right ovary at the first session and the left ovary at the fifth session (*p* = 0.9982). In pubertal heifers, *HAS3* expression in the right ovary at the fifth session was higher than at the first session and than in the left ovary at the fifth session (both *p* < 0.0001), with no difference between the right ovary at the first session and the left ovary at the fifth session (*p* = 0.3525). No differences were observed between pre‐pubertal and pubertal heifers at the first OPU session or between control ovaries at the fifth session (Figure 3B).

*MMP2* expression showed opposite patterns between the two age groups. In pre‐pubertal heifers, expression in the right (punctured) ovary at the fifth OPU session was higher than at the first session and than in the left (control) ovary at the fifth session (both *p* ≤ 0.0002), whereas no difference was observed between the right ovary at the first session and the left ovary at the fifth session (*p* = 0.7870). In pubertal heifers, *MMP2* expression in the right ovary at the fifth session was lower than at the first session and than in the left ovary at the fifth session (both *p* < 0.0001), whereas no difference was observed between the right ovary at the first session and the left ovary at the fifth session (*p* > 0.9999). No difference between age groups was observed at the first OPU session (Figure 3C).

For *TIMP2*, expression in pre‐pubertal heifers was higher in the right (punctured) ovary at the fifth OPU session than at the first session and than in the left (control) ovary at the fifth session (both *p* ≤ 0.0016). Expression in the right ovary at the first session and in the left ovary at the fifth session was lower than in the corresponding pubertal conditions (both *p* ≤ 0.0018). In pubertal heifers, *TIMP2* expression did not differ among the right ovary at the first session, right ovary at the fifth session, and left ovary at the fifth session (Figure 3D).

#### 3.3.2 Genes Related to Follicular Activation and Ovarian Function

In pre‐pubertal heifers, *PTEN* expression in the right (punctured) ovary at the fifth OPU session was higher than at the first session and in the left (control) ovary at the fifth session (both *p* < 0.0001), whereas no difference was observed between the right ovary at the first session and the left ovary at the fifth session (*p* = 0.9995). A similar pattern was observed in pubertal heifers, with higher *PTEN* expression in the right ovary at the fifth session than at the first session and in the left ovary (both *p* < 0.0001), while no difference was found between the right ovary at the first session and the left ovary at the fifth session (*p* = 0.9417). The increase in *PTEN* expression between the first and fifth OPU sessions differed between pre‐pubertal and pubertal heifers. No differences were observed between the two age groups at the first OPU session or between the control ovaries at the fifth session (*p* > 0.05) (Figure 4A).

**FIGURE 4:**
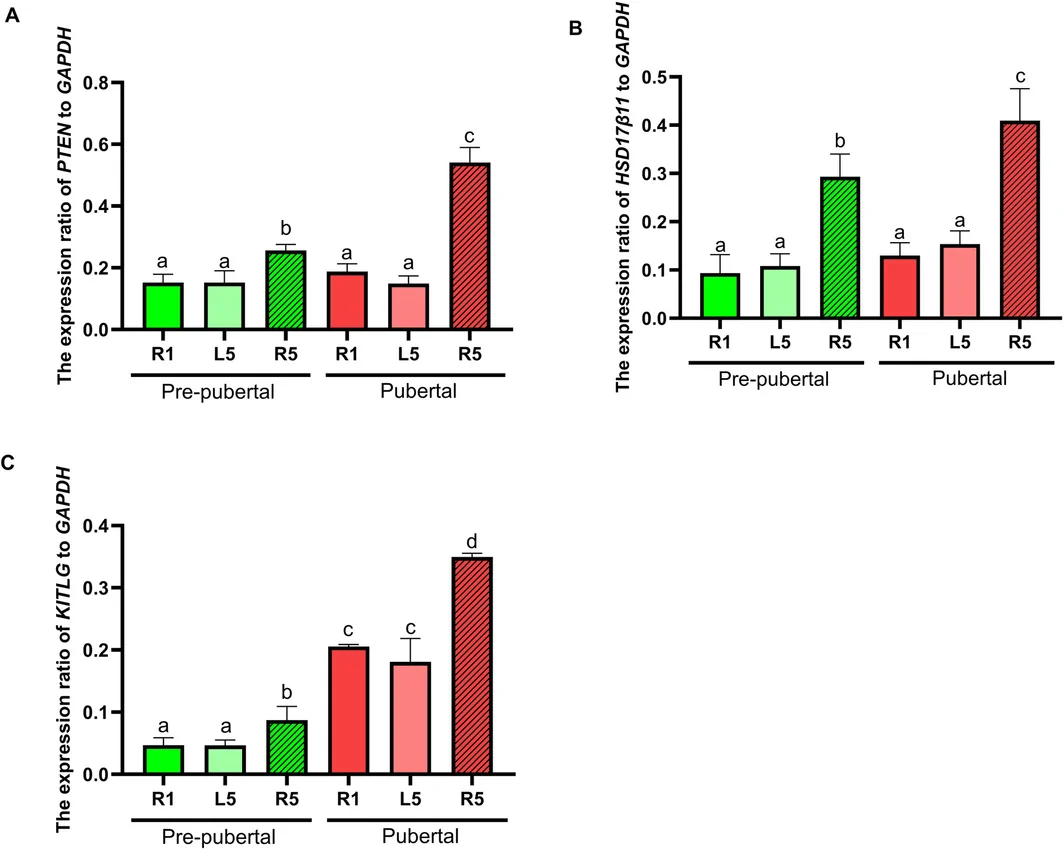
Expression of (A) *PTEN*, (B) *HSD17β11*, and (C) *KITLG* mRNA in ovarian tissue of pre‐pubertal and pubertal heifers subjected to five OPU sessions. Data are presented as mean ± SEM. Differences between compared conditions are indicated by different lowercase letters (a, b, c; *p* < 0.05). L5, left (control, non‐punctured) ovary at the fifth OPU session; R1, right (punctured) ovary at the first OPU session; R5, right (punctured) ovary at the fifth OPU session.

A similar pattern was observed for *HSD17β11*. In pre‐pubertal heifers, expression in the right (punctured) ovary at the fifth OPU session was higher than at the first session (*p* = 0.0061) and in the left (control) ovary at the fifth session (*p* < 0.0001), whereas no difference was observed between the right ovary at the first session and the left ovary at the fifth session (*p* = 0.9973). In pubertal heifers, *HSD17β11* expression in the right ovary at the fifth session was higher than at the first session and in the left ovary (both *p* < 0.0001), while no difference was observed between the right ovary at the first session and the left ovary at the fifth session (*p* = 0.9941). The increase in expression between the first and fifth OPU sessions was lower in pre‐pubertal than in pubertal heifers (Figure 4B).

*KITLG* expression showed a similar pattern. In pre‐pubertal heifers, expression in the right (punctured) ovary at the fifth OPU session was higher than at the first session (*p* < 0.0001) and in the left (control) ovary at the fifth session (*p* = 0.0100), whereas no difference was observed between the right ovary at the first session and the left ovary at the fifth session (*p* = 0.9987). In pubertal heifers, *KITLG* expression in the right ovary at the fifth session was higher than at the first session and in the left ovary (both *p* < 0.0001), while no difference was observed between the right ovary at the first session and the left ovary at the fifth session (*p* = 0.5917) (Figure 4C).

## 4 Discussion

The present study shows that five OPU sessions were associated with alterations in follicular dynamics and the expression of genes related to ECM remodelling and follicular regulation. These findings indicate that, under the experimental conditions of the present study, five OPU sessions were associated with local ovarian responses, including changes in follicle number and gene expression, without detectable changes in systemic serum E2 and AMH concentrations. Variation in follicle number associated with OPU sessions has already been described for *Bos indicus* cows (Viana et al. 2010). Similarly, repeated follicular puncture in dairy heifers was reported to cause no major impairment of subsequent ovarian function (Petyim et al. 2000). The transient increase in follicle number during the fourth session is consistent with a local ovarian response to repeated puncture, without detectable changes in systemic hormone concentrations. These findings are consistent with previous studies showing that increased OPU frequency can increase the number of medium‐sized follicles and improve oocyte quality and developmental competence (Saleem et al. 2022). Such effects may reflect local tissue adaptation involving ECM remodelling and mechanotransduction pathways. Consistent with this hypothesis, previous studies have shown that localized mechanical stress can induce cellular responses that promote follicular growth (Shah et al. 2018). Structural differences between pre‐pubertal and pubertal ovaries, including collagen fibre thickness, elastin content, and microfibrillar protein composition, may modulate how the cortex responds to mechanical stress (Ouni et al. 2020).

The observed upregulation of *COL3A1* in punctured ovaries of both pre‐pubertal and pubertal heifers was associated with local ovarian responses following ovarian puncture and may reflect changes related to tissue remodelling and repair. The higher baseline expression in pubertal animals further indicates that collagen deposition increases with reproductive maturity, consistent with reports of age‐ and activity‐dependent accumulation of collagens in the ovary (Ouni et al. 2020). Changes in collagen expression could potentially contribute to alterations in the mechanical properties of the ovarian microenvironment. *HAS3* gene expression increased in the punctured right ovary after the fifth OPU session in both pre‐pubertal and pubertal heifers, while the left ovary remained unchanged. This local response may be associated with changes in ECM organization following ovarian puncture. *HAS3* contributes to hyaluronan synthesis and ECM organization, and changes in its expression may influence the local ovarian microenvironment (Parkes et al. 2021). However, the functional consequences of the observed HAS3 upregulation remain to be established. *MMP2* and *TIMP2* exhibited complementary patterns depending on ovarian maturity. In pre‐pubertal heifers, increased *MMP2* and *TIMP2* expression was observed during the fifth session of ovarian puncture, suggesting a coordinated transcriptional response involving ECM‐related genes following puncture. Conversely, in pubertal heifers, decreased *MMP2* and stable *TIMP2* expression were observed during the fifth session, indicating a more limited transcriptional response to ovarian puncture in pubertal ovaries. In pre‐pubertal heifers, the coordinated increase in *MMP2* and *TIMP2* may be consistent with altered regulation of ECM turnover following repeated puncture, whereas the more limited response observed in pubertal heifers may indicate differences in the regulation of ECM turnover between developmental stages (Peralta et al. 2017).

The local ovarian response observed in the present study may reflect not only the consequences of follicular puncture itself, but also tissue injury and subsequent inflammatory and repair processes. In the ovary, tissue injury associated with ovulation is accompanied by a local inflammatory response and subsequent wound healing (Papacleovoulou et al. 2011). Repeated follicular puncture has also been associated with ovarian tissue injury, including inflammation and stromal fibrosis (Malard et al. 2020). Malard et al. (2020) distinguished between an acute OPU model, involving four OPU sessions, and a chronic model represented by cows with a prolonged history of repeated OPU and reduced reproductive performance. In the present study, five OPU sessions were performed at seven‐day intervals without hormonal stimulation. Therefore, the observed changes likely reflect a local ovarian response to repeated puncture rather than chronic ovarian damage. The observed changes in ECM‐related gene expression may reflect local tissue repair and remodelling following repeated ovarian injury. However, the present data do not allow us to determine whether inflammatory or fibrotic processes were involved.

*PTEN*, which encodes a negative regulator of the PI3K/AKT pathway, was upregulated following five OPU sessions, with a more pronounced increase in pubertal than in pre‐pubertal heifers. Given its role in follicular regulation, this increase may reflect modulation of PI3K/AKT signalling. Inhibition of *PTEN* has been shown to promote primordial follicle activation, whereas prolonged activation of the PI3K/AKT pathway may negatively affect follicle and oocyte quality (Maidarti et al. 2019). However, the functional significance of the observed changes in PTEN expression cannot be determined from the present gene expression data alone. *KITLG* plays a crucial role in ovarian folliculogenesis, supporting oocyte competence and follicle survival through paracrine signalling mechanisms (Lussier et al. 2017). In the present study, *KITLG* expression increased locally in the punctured ovary in both pre‐pubertal and pubertal heifers, with a less pronounced response in pre‐pubertal animals. No changes were observed in the non‐punctured ovaries, consistent with a localized ovarian response to repeated puncture. This finding is consistent with previous evidence linking *KITLG* expression with follicle maturation and oocyte competence (Lussier et al. 2017), although its functional significance in the present context remains to be determined. In the present study, *HSD17β11* mRNA increased after five OPU sessions, with a stronger response observed in pubertal heifers. This finding contrasts with previous in vitro results in bovine ovarian cortical cultures, where *HSD17β11* expression was downregulated during insulin‐induced follicle activation (Yang and Fortune 2015), possibly reflecting differences between experimental models.

Several limitations should be considered when interpreting the present findings. The study was based on gene expression analysis without protein‐level or functional validation; therefore, the observed changes should not be interpreted as direct evidence of functional ECM remodelling, mechanotransduction, or follicular activation. Histological assessment of inflammatory and fibrotic responses was also not performed, limiting interpretation of the potential contribution of tissue injury, inflammation and wound healing. Further studies integrating molecular, histological and functional analyses are warranted to clarify these local ovarian responses.

## 5 Conclusion

Five OPU sessions were associated with local changes in follicle number and in the expression of genes related to extracellular matrix remodelling, follicular regulation and intracellular signalling in pre‐pubertal and pubertal heifers. These responses occurred without detectable changes in systemic serum E2 and AMH concentrations or ovarian area, supporting the presence of localized ovarian responses under the experimental conditions of the present study. The magnitude and pattern of gene expression changes differed between pre‐pubertal and pubertal heifers, indicating developmental differences in the ovarian response to five OPU sessions.

## Author Contributions

S.G.: investigation, methodology, formal analysis, visualization, writing – original draft. I.K.‐Z.: conceptualization, supervision, methodology, formal analysis, writing – original draft, writing – review and editing. D.B.: conceptualization. I.W.‐P.: conceptualization, investigation, writing – review and editing. All authors read and approved the final manuscript.

## Funding

This research was funded by the National Science Center in Poland (2021/41/B/NZ9/03661).

## Ethics Statement

All animal procedures were approved by the Local Animal Care and Use Committee of Olsztyn (Agreement No. 55/2023) and were conducted in accordance with relevant European Union and Polish regulations and guidelines concerning the protection and use of animals for scientific purposes.

## Conflicts of Interest

The authors declare no conflicts of interest.

## Data Availability

All data generated or analysed during this study are available from the corresponding author on reasonable request.
